# An intra-annual 30-m dataset of small lakes of the Qilian Mountains for the period 1987–2020

**DOI:** 10.1038/s41597-023-02285-x

**Published:** 2023-06-07

**Authors:** Chao Li, Shiqiang Zhang, Dahong Zhang, Gang Zhou

**Affiliations:** 1 College of Urban and Environmental Science, Northwest University, Xi’an, 710127 P.R. China; 2 Shaanxi Key Laboratory of Earth Surface System and Environmental Carrying Capacity, Northwest University, Xi’an, 710127 P.R. China

**Keywords:** Limnology, Hydrology

## Abstract

Small lakes (areas between 0.01 km^2^ and 1 km^2^) on the Qinghai–Tibet Plateau (QTP) are prone to fluctuations in number and area, with serious implications for the surface water storage and water and carbon cycles of this fragile environment. However, there are no detailed long-term datasets of the small lakes of the QTP. Therefore, the intra-annual changes of small lakes in the Qilian Mountains region (QMR) in the northeastern part of the QTP were investigated. The small lake water bodies (SLWB) in the QMR were extracted by improving existing commonly used waterbody extraction algorithms. Using the Google Earth Engine platform and 13,297 Landsat TM/ETM + /OLI images, the SLWB of the QMR were extracted from 1987 to 2020 applying the improved algorithm, cross-validation and manual corrections. The reliability, uncertainty and limitations of the improved algorithm were discussed. An intra-annual small lake dataset for QMR (QMR-SLD) from 1987 to 2020 was released, containing eight attributes: code, perimeter (km), area (km^2^), latitude and longitude, elevation (m), area error, relative error (%), and subregion.

## Background & Summary

Lakes are important components of terrestrial water resources, closely related to the lithosphere, atmosphere, and biosphere, and supporting economic activities, including industrial and agricultural production, biodiversity, commercial activities, and human health^1,2^. The Qinghai-Tibet Plateau (QTP) hosts an abundance of alpine lakes that are strongly influenced by the melting of the cryosphere (i.e., glaciers and snow) and climate change^3,4^. In recent decades, many QTP lakes with areas larger than 1 km^2^ have shown drastic changes, such as a significant increase in number, area, lake storage, and a rapid rise in water levels^4,5^.

Compared with many large lakes on the QTP, small lakes (areas between 0.01 km^2^ and 1 km^2^) are characterized by wide distribution, gentle basin slopes and complex morphologies. In addition, due to poorly developed drainage channels, most of these lakes have a closed flow, with shallow waters and rapid evaporation^6^. As a result, small lakes are more likely to change rapidly in number and area, with severe impacts on the highland’s water cycle, surface water storage, and fragile permafrost ecosystems^7,8^. Additionally, small lakes contribute significantly to lake systems in terms of primary productivity, biodiversity and carbon cycling^9^. Among the small lakes on the QTP (typically less than 0.5 km^2^ ^10^), thermokarst lakes have been found to be sources of methane release with significant seasonal and spatial variability, typically peaking during lake ice melt^11^. Therefore, monitoring changes in alpine small lake water bodies (SLWB) in the QTP is important for managing water strategies, predicting flood events, and developing sustainable regional management plans.

Most of the existing datasets and studies quantifying changes in lake number and area on the QTP are about medium to large lakes (larger than 1 km^2^) with well-defined boundaries^5,12,13^. The area and spatio-temporal characteristics of small lakes have been described for some regions^14,15^. There are more than ten published datasets related to small lakes for the QTP or its sub-regions. For example, Wang *et al*.^14^ published glacial lake datasets (0.0054‒6.46 km^2^) for the alpine region of Asia at 30-m resolution for 1990 and 2018; Chen *et al*.^16^ published an annual dataset of glacial lakes (>0.0081 km^2^) in the QTP from 2008 to 2017 based on Landsat data at 30-m resolution; Dou *et al*.^15^ created a multi-temporal inventory of QTP glacial lakes (>0.0081 km^2^) from 1990 to 2019. The above datasets and related studies on small lakes are all dominated by glacial lakes. However, the small lakes of the QTP include not only glacial lakes but also a large number of thermokarst and other types of lakes. The temporal resolution of current datasets monitoring small lakes of the QTP does not allow tracking intra-annual changes in small lakes, limiting the understanding of complex fluctuations in SLWB due to climatic factors such as precipitation.

A large number of images are needed to continuously monitor the SLWB for a long period with high temporal and spatial resolution^6,8^. Remote sensing data of the Landsat series have been widely used for lake water monitoring due to their longest satellite data record and medium spatial resolution. The Google Earth Engine (GEE) platform integrates common remote sensing datasets (e.g., Landsat, MODIS, and Sentinel-2) and enables online visualization and computational analysis processing of these datasets, lowering research costs by eliminating tedious preliminary work^17,18^. Therefore, Landsat data can be used to monitor the dynamics of SLWB with the GEE platform.

The extraction of SLWB in the QTP needs a robust algorithm with high accuracy. The extraction of water bodies from optical images using remote sensing is commonly achieved by determining a threshold of a water body index^19^. Common water body indexes include the Normalized Difference Water Index (NDWI)^20^, Modified NDWI (MNDWI)^21^, and Automatic Water Extraction Index (AWEI)^22^. Although NDWI and MNDWI are popular for their ability to accurately identify water bodies^22,23^, they have two main limitations. Firstly, NDWI and MNDWI are not sufficiently sensitive to mixed pixels of vegetation and water bodies to accurately extract water bodies in vegetation-covered areas^24^. Secondly, it is difficult to select the thresholds for NDWI and MNDWI and they have a large effect on the accuracy of the water body extraction results. The widely used threshold segmentation algorithm OTSU^25^ has its drawbacks, such as the difficulty in ensuring the accuracy of the results of local water extraction in large catchments^26^. The optimal threshold can also be established by visual interpretation. However, visual interpretation is prone to human subjective errors and is time-consuming. Alternatively, water can be accurately extracted from optical satellite data by establishing a relationship between the MNDWI, normalized difference vegetation index (NDVI) and enhanced vegetation index (EVI)^22,27^. The water body extraction algorithm, MNE, is defined as ((MNDWI > NDVI or MNDWI > EVI) and (EVI < 0.1)), which was proposed by Zou *et al*.^27^. This algorithm has been widely applied to extract water bodies with satisfactory accuracy^28^. Its main advantage is that there is no need to pick a threshold for each image. Additionally, Zhou *et al*.^22^ proposed an improved algorithm, IMNE, defined as (MNDWI > NDVI or MNDWI > EVI), for extracting water bodies in the Yellow River source area. This algorithm increases the robustness of the extraction results compared to the MNE. However, this approach does not always extract water bodies effectively^22^, especially in highland regions^29^, and needs to be improved for the accurate extraction of SLWB.

The Qilian Mountains region (QMR) is situated in the northeastern QTP. The southern slope of the QMR is a crucial water supply region for Qinghai Lake and the Yellow River. The northern slope of the QMR is the birthplace of China’s inland rivers (e.g., the Heihe, Shiyang and Shule rivers), which are the main sources of freshwater and critical for the stability of the region. Thus, the Chinese scientific community calls this region the “Wet Island of China” and the “Alpine Water Tower”^30^. The SLWB in the QMR has undergone large changes under the impact of climate variation and the intensification of human activities. This paper takes the QMR on the QTP as the study area. The specific objectives of this study are to: (1) improve the water body extraction algorithm of MNE to obtain a more accurate and robust SLWB extraction algorithm for the QMR; (2) publish the QMR small lakes dataset based on consecutive Landsat TM/ETM + /OLI from 1987 to 2020 and our improved water extraction algorithm; (3) explore the reliability, uncertainties and limitations of the QMR SLWB dataset, as well as to provide an outlook on future small lake studies for the QTP; (4) analyze the spatial and temporal dynamics of SLWB in the QMR. This study fills the gap in intra-annual SLWB data from 1987 to 2020 in the QMR.

## Methods

### Study area

**Fig. 1:**
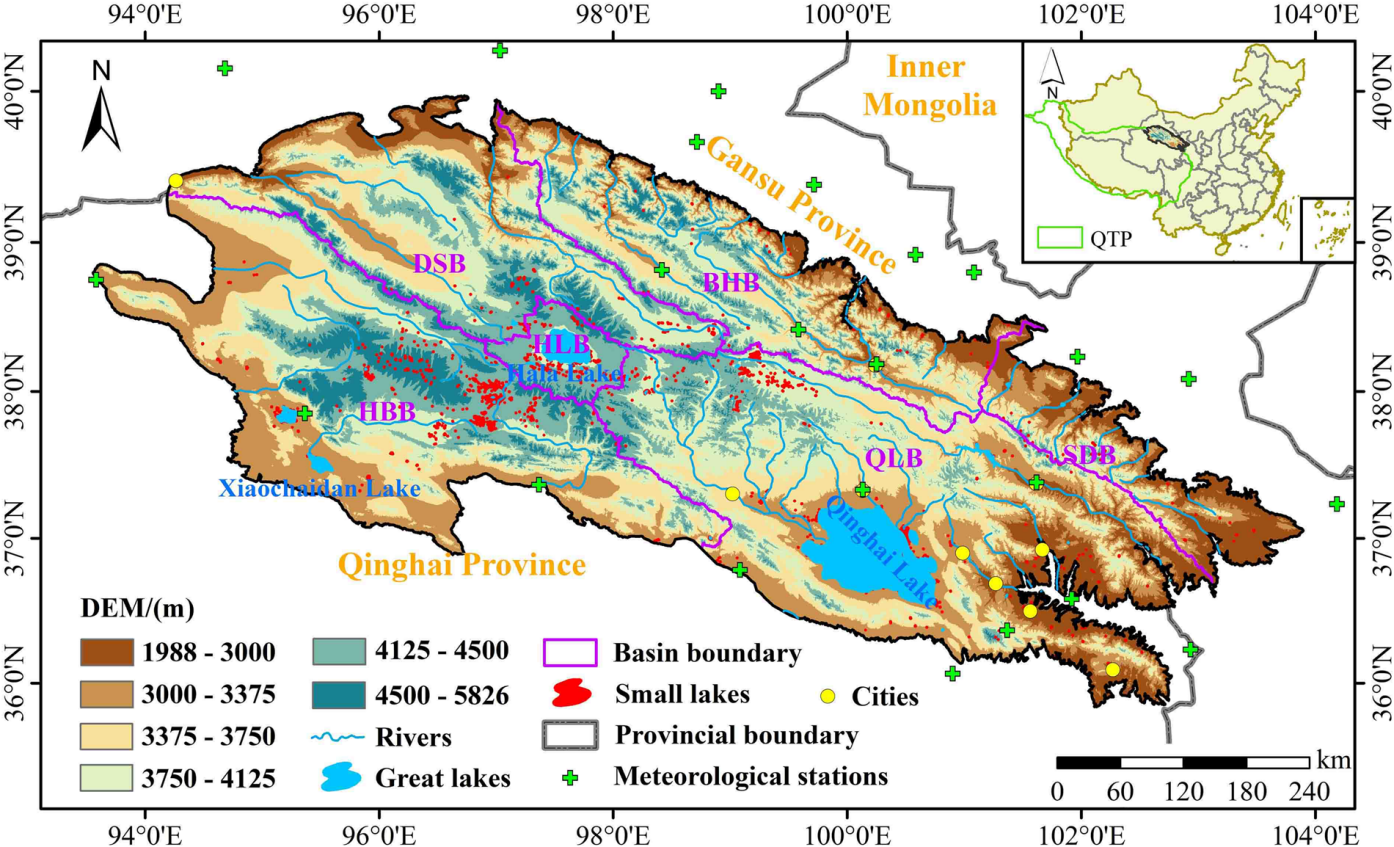
Geographical location and topography of the QMR.

The QMR is situated in the northeastern QTP (Fig. 1). The area of the study region is about 3.10 × 10^5^ km^2^. The elevation is mostly above 3000 m a.s.l. and gradually decreases from southwest to northeast. The geomorphological structure is complex and variable^30^. The QMR has a typical continental plateau climate^31^. The eastern part of the QMR has high humidity and more precipitation, while the western part is dry with little rainfall^30^, with an annual precipitation of 200~500 mm mainly concentrated in the summer. The annual mean air temperature is low (<2 °C). The annual and daily temperature are highly variable in space. The water system of the QMR is dominated by glacial meltwater replenishment and mountain precipitation, with a radial-grid distribution and drainage from northwest to southeast^32^. The natural ecosystem is fragile and sensitive to climate variations^33^. To study the water dynamics of small lakes at different regional scales in the QMR, we utilized the Level 4 sub-basin information provided by HydroSHEDs database^34^ (https://www.hydrosheds.org/, last access: 5 October 2021) to divide the entire QMR into six basins after minor adjustments. HydroSHEDs database has been widely used because of its high reliability^28,35^. The six basins include the Qinghai Lake Basin, Hala Lake Basin, Shiyang River & Datong River Basin, Danghe River & Shule River, Beida River & Heihe River Basin, Haleteng River & Bayinguole River Basin.

### Source data

**Fig. 2:**
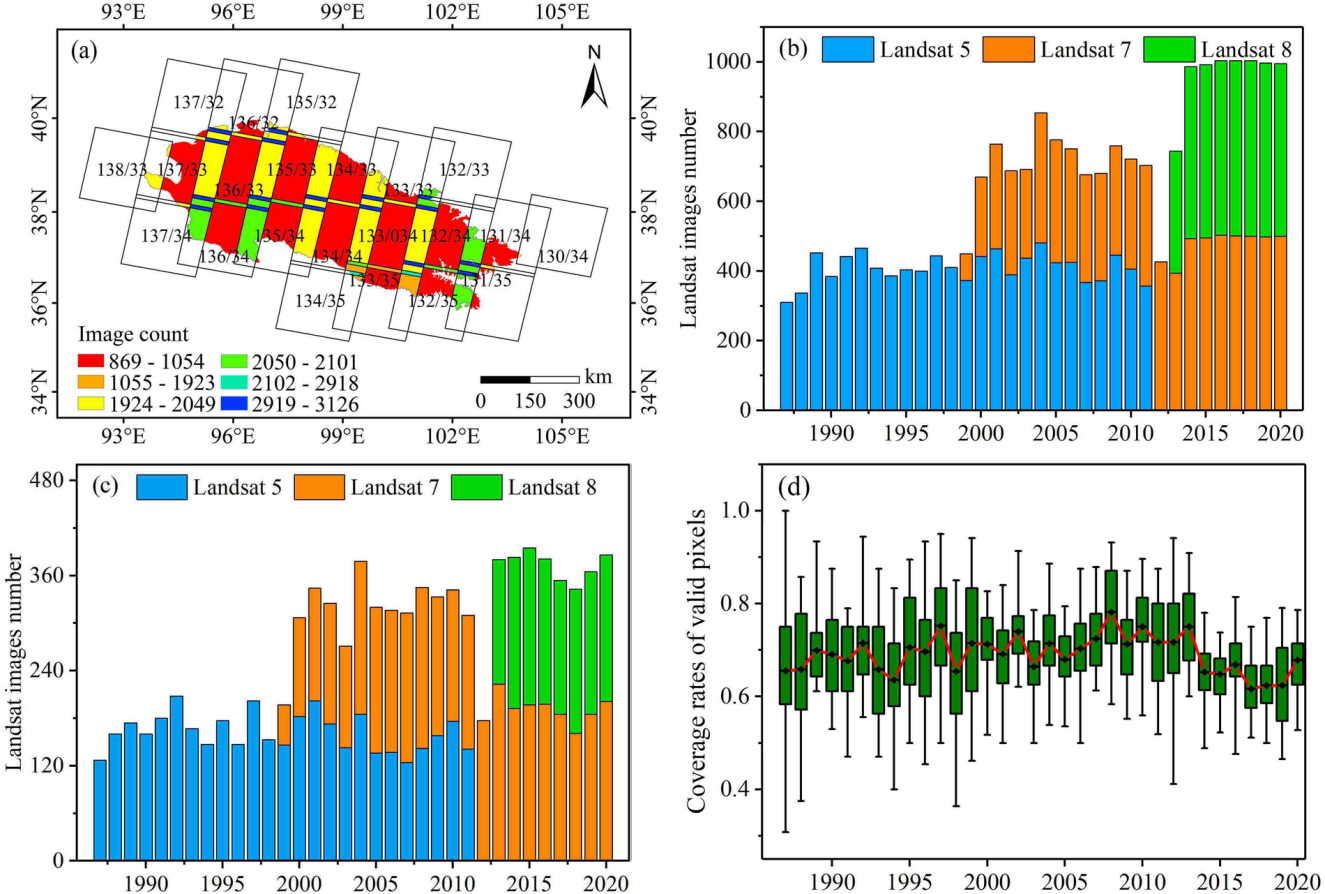
Distribution of Landsat data used in the study. (**a**) Number of images covered by each Landsat path number for the QMR from 1987 to 2020; (**b**) Total number of Landsat images covering the study area each year; (**c**) Number of Landsat images with less cloud cover for each year; (**d**) Percentage of all high-quality pixel observations.

**Fig. 3:**
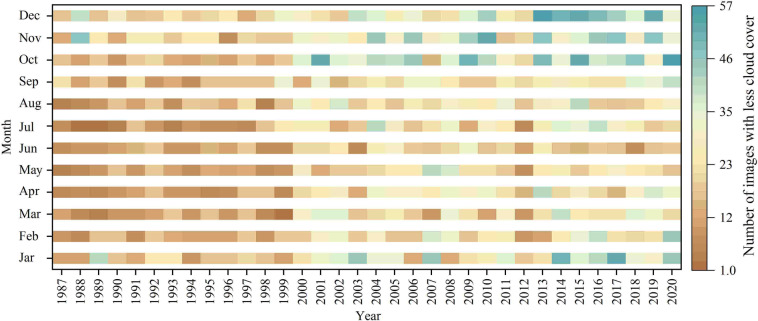
Temporal stages of remote sensing images selected for SLWB extraction in the QMR.

The intra-annual SLWB of the QMR during 1987‒2020 were extracted using Landsat surface reflectance data^36^ with cloud cover of less than 10%. The number of Landsat images and the percentage of all high-quality pixels without cloud, cloud shadow or snow coverage are shown in Fig. 2. The number of these high-quality Landsat images was 13,297 (>12 TB of data). The average rate of valid pixels (ratio of the number of pixels without cloud coverage to the total number of pixels) was high (69%) throughout the study period (Fig. 2d), which satisfied the requirement of SLWB extraction. It means that the extraction accuracy of SLWB is reliable and further able to satisfy the delineation of permanent, seasonal and ephemeral water bodies. We also counted the number of images with low-coverage clouds or cloud shadows for each month between 1987 and 2020 (Fig. 3). The temporal coverage of the images with low cloud cover was adequate for the later delineation of different types of SLWB. All Landsat image processing tasks were performed on the GEE platform. The global surface water (GSW) dataset^37^ with a 30-m resolution on the GEE platform, produced by the Joint Research Center, was employed to verify the precision of various water body types extracted using three different algorithms in this study. ALOS World 3D-30m (AW3D30)^38^, a global digital surface model (DSM) dataset with a 30-m resolution available on the GEE platform, was used to remove mountain shadows and calculate the elevation of small lakes. Forty-four Sentinel-2A images with the same obtained date as the Landsat images were used to verify the accuracy of the three SLWB extraction algorithms derived from Landsat data (Table S1). The Sentinel-2A data were acquired from the GEE platform. Cloud and cloud shadow pixels were removed from all Sentinel-2A images. Glacier data^39^ were downloaded from the Institute of Tibetan Plateau Research Chinese Academy of Science (https://data.tpdc.ac.cn/home, last access: 20 October 2022). The Global River Widths from Landsat database^40^ and HydroSHEDs database^34^ were used to mask rivers. The Global River Widths from Landsat database was downloaded from https://zenodo.org/record/1297434#.YrvEzj5ByUk (last access: 12 October 2021).

### Method flow

**Fig. 4:**
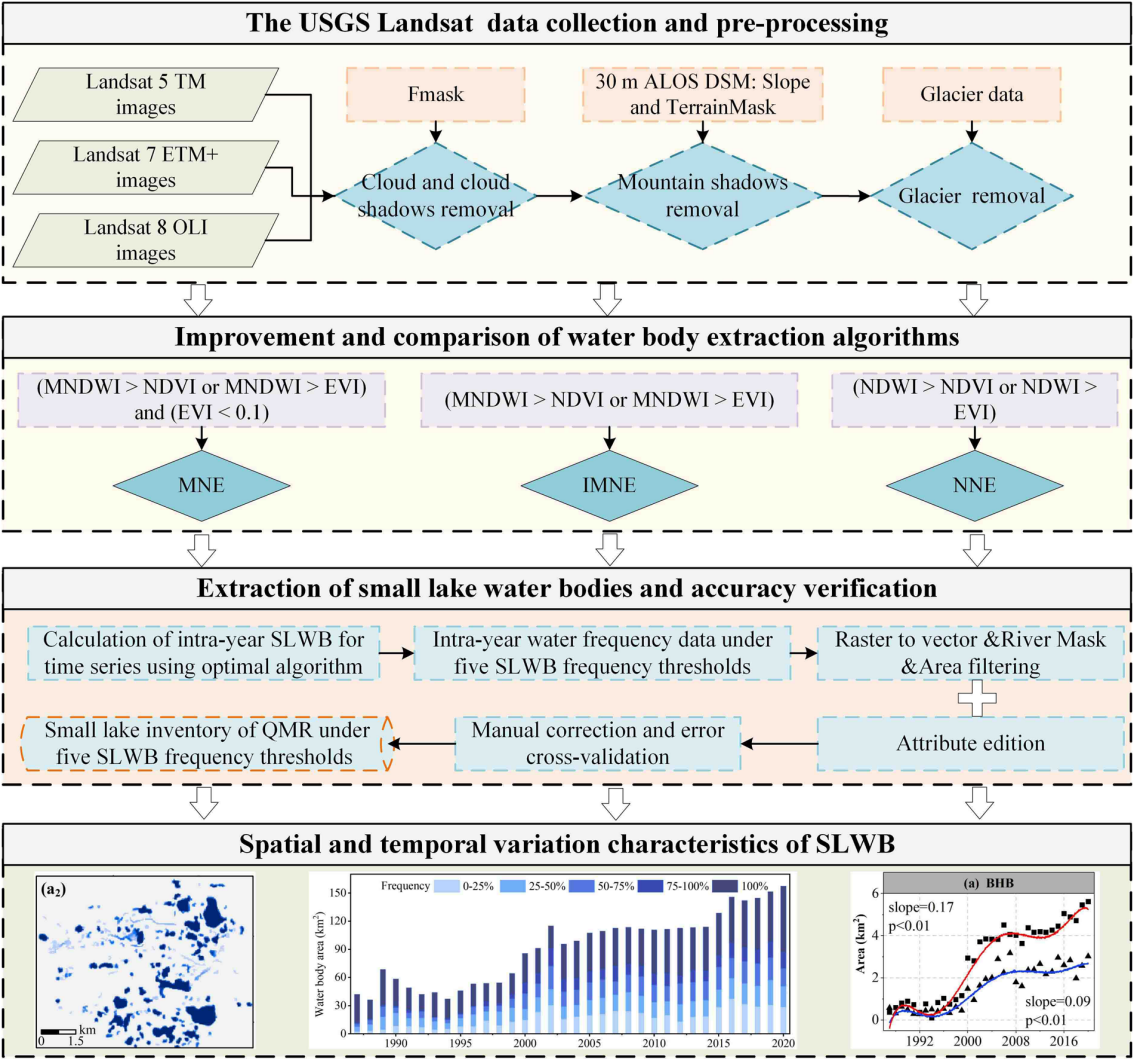
Workflow of the study to identify the SLWB dataset in the QMR.

The specific steps for SLWB extraction from the Landsat images include (1) removing clouds and cloud shadows using the Function of the Mask (Fmask) (see Zhu and Woodcock^41^ and Zhu *et al*.^42^ for a detailed description of the mask); (2) Removal of non-water objects with slopes greater than 7 degrees and topographic shading of less than 150 degrees using ALOS DSM data^43^. Considering differences in time acquisitions between ALOS DSM data and Landsat images, the calculated topography may not exactly match the actual topography, resulting in minor errors when masking small lakes. These errors were subsequently corrected manually and through cross-validation; (3) Removing glacier using the glacier data; (4) Comparison and validation of the three water extraction algorithms using a confusion matrix^28^ to obtain the optimal algorithm for SLWB; (5) Calculation of intra-annual SLWB using an improved water body extraction algorithm (see the following water extraction algorithm section for details) for time-series Landsat images and extraction of intra-annual water frequency data under five SLWB frequency thresholds; (6) Removal of extracted non-lake water bodies using vector data derived from the Global River Widths from Landsat dataset and HydroSHEDs dataset; (7) Manual inspection and refinement of individual small lakes and addition of the associated eight attributes (code, latitude and longitude, perimeter, area, elevation, area error, relative error and subregion) for each lake. The complete description of the code can be found in Wang *et al*.^14^. Automated processing using GEE was followed by strict quality control with visual checks and correction of mapping errors to ensure quality; (8) Analysis of the spatio-temporal characteristics of the SLWB of the QMR. The workflow of the whole study is shown in Fig. 4.

### Water extraction algorithm

Currently, there is no agreement on the size range for small lakes. We have established the lower limit for small lakes to be 0.01 km^2^ ^7,44^ based on Landsat TM/ETM + /OLI data used in this study. Extracting small lakes with areas less than 0.01 km^2^ is difficult to guarantee due to spatial resolution limitations of Landsat data^44^. Recent research by Pi *et al*.^9^ guided us to set the upper limit threshold for small lakes at 1 km^2^. Therefore, we have delineated the size range for small lakes of SLWB as 0.01 km^2^ to 1 km^2^.

**Fig. 5:**
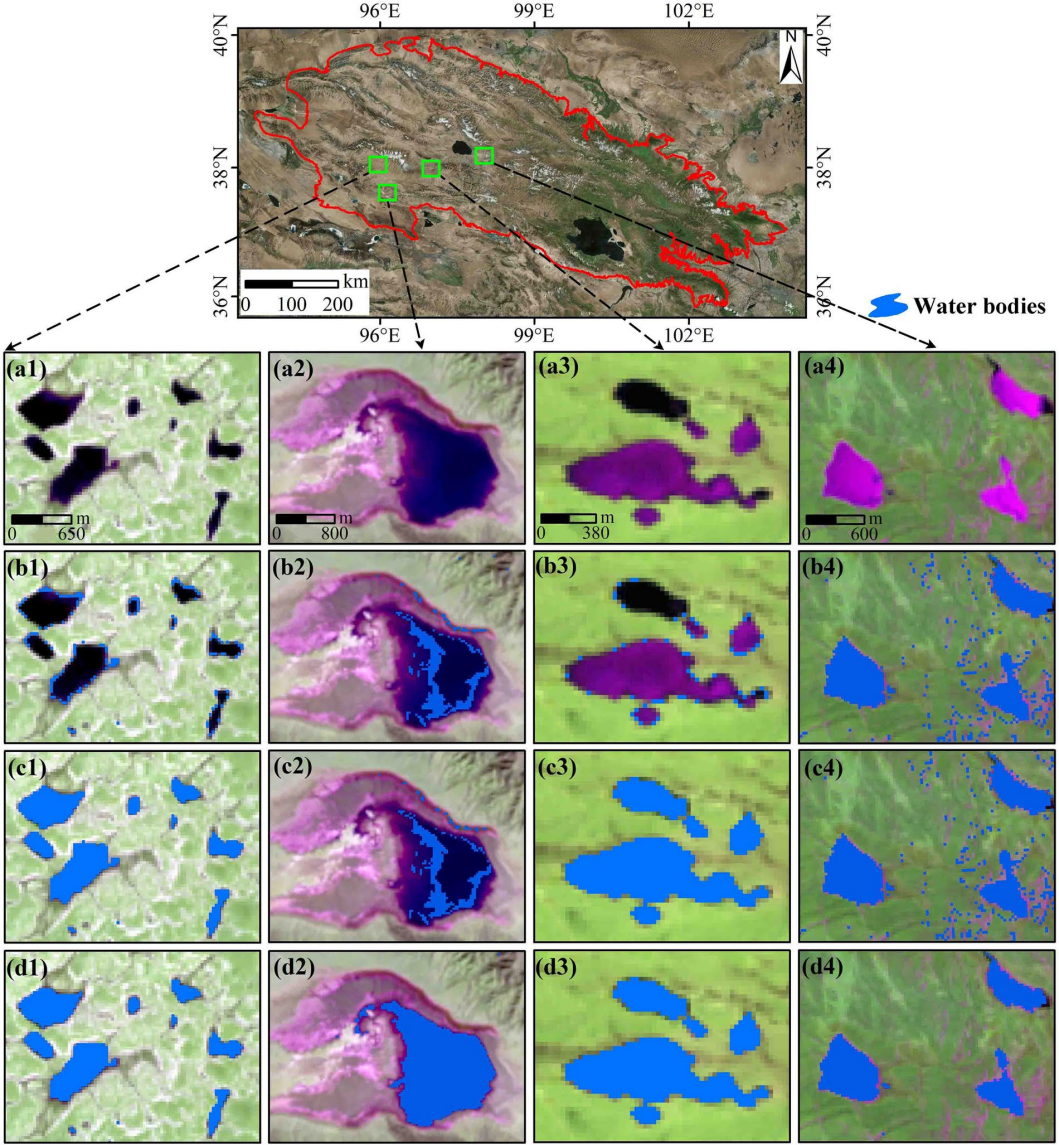
Validation of the lake extraction method (a1-a4 are the false-color images, b1-b4 are the SLWB extraction results based on the MNE, c1-c4 are the SLWB extraction results using the IMNE, and d1-d4 are the SLWB extraction results based on the NNE).

The MNE proposed by Zou *et al*.^27^ has been widely used to extract water bodies^23,28,45^. However, Zhou *et al*.^22^ and Chen *et al*.^46^ found that the accuracy of the MNE varies widely for different regions, and thus is not applicable for extracting water bodies in all regions. In particular, MNE is less effective in extracting water bodies in highland mountains^22,29^. Worden and de Beurs^29^ found that the overall accuracy of MNE for extracting water bodies containing vegetation in the Caucasus was only 78%. Zhou *et al*.^22^ found that the problem was primarily caused by EVI < 0.1 and made improvements to the MNE. When the MNE changed to (MNDWI > NDVI or MNDWI > EVI) (IMNE), the extraction results of the Yellow River source water bodies were significantly improved. However, we found that the above two methods were not suitable for extracting surface water bodies in the QMR, and the extracted SLWB was sometimes missing (Fig. 5). After many trials, we found that the SLWB extraction was significantly ameliorated when the MNDWI in IMNE was replaced with the NDWI to form the new method (NDWI > NDVI or NDWI > EVI) (NNE). This is due to the relatively better performance of NDWI compared to MNDWI for lake water monitoring across the QTP^5^. Compared to MNDWI, NDWI is more effective in accurately mapping lake areas by providing a clear outline of the water body^47^. It can even delineate lake boundaries with clarity, even under cloud cover. Furthermore, NDWI is capable of providing clear delineation of lake boundaries even under cloud cover, making it a more reliable tool for mapping water bodies than MNDWI^47^. Therefore, we extracted the SLWB range using the NNE.

1 $$MNDWI=\frac{{\rho }_{green}-{\rho }_{swir1}}{{\rho }_{green}+{\rho }_{swir1}}$$

2 $$NDVI=\frac{{\rho }_{nir}-{\rho }_{red}}{{\rho }_{nir}+{\rho }_{red}}$$

3 $$EVI=2.5\times \frac{{\rho }_{nir}-{\rho }_{red}}{{\rho }_{nir}+6\times {\rho }_{red}-7.5\times {\rho }_{blue}+1}$$

4 $$NDWI=\frac{{\rho }_{green}-{\rho }_{nir}}{{\rho }_{green}+{\rho }_{nir}}$$

where *ρ*_*green*,_ *ρ*_*swir1*_, *ρ*_*nir*_, *ρ*_*red*_, and *ρ*_*blue*_ are the green, short-wave infrared, near-infrared, red, and blue bands of the Landsat images, respectively.

### Verification of three SLWB extraction algorithms

We randomly selected 12,000 test samples from 44 Sentinel-2A images (Table S1), which included 6,612 water samples and 5,388 non-water samples. To ensure the reliability of the evaluation, some water body samples were selected in the transition zone of shallow and deep lakes, while some non-water samples were selected over land areas close to a water body (such as lakeshores and lake islands). The test samples were then visually interpreted. Finally, the pixels of the 12,000 samples in the Landsat images were compared for consistency with the water body information of the decoded Sentinel-2A images.

The confusion matrix for the assessment of three water body extraction algorithms is shown in Table 1. The results indicate that the overall accuracy and Kappa coefficient of the NNE are 98.14% and 0.96, respectively, indicating that the extracted water body by NNE has higher accuracy compared to MNE and IMNE and can be used for further extraction of water body information in time series.

**Table 1:** Confusion matrix for accuracy assessment of water body extraction algorithms.

Method		Samples	Sentinel		Total	User accuracy	Overall accuracy	Kappa coefficient
			Water	Non‒water				
MNE	Landsat	Water	6561	862	7423	0.8838	0.9239	0.8440
		Non‒water	51	4526	4577	0.988		
		Total	6612	5388	12000			
	Total producer’s accuracy		0.9923	0.8402				
IMNE	Landsat	Water	6580	766	7346	0.8957	0.9335	0.8638
		Non‒water	32	4622	4654	0.9931		
		Total	6612	5388	12000			
	Total producer’s accuracy		0.9953	0.8579				
NNE	Landsat	Water	6513	124	6637	0.9813	0.9814	0.9624
		Non‒water	99	5264	5363	0.9815		
		Total	6612	5388	12000			
	Total producer’s accuracy		0.9851	0.9770				

### Calculation and classification of SLWB

There are significant seasonal variations in the lake boundaries of the QTP^48^. Smaller lakes are more likely to change boundaries^49^. Additionally, Landsat data are highly influenced by clouds and cloud shadows, making images with large amounts of cloud and cloud shadows unusable. Landsat images with less than 10% clouds were selected and de-clouded and de-shadowed using the Fmask algorithm. This resulted in a significant reduction in the actual available temporal resolution of Landsat data. In addition, the large study area was covered by several satellite tracks, which differed considerably in acquisition times and quantity of high-quality images. This causes the extracted SLWB to miss some small lakes and makes it difficult to capture their seasonal variations on the same date.

Therefore, we used the water body frequency approach with different thresholds^28^ to extract the intra-annual SLWB from Landsat data. This method takes advantage of time series of Landsat images with the GEE platform and reduces the potential errors due to image quality and water body extraction algorithms uncertainties, making the extracted SLWB very robust^48,50^. The approach has been shown to be reliable and effective for obtaining intra-annual lake water bodies^48,51^. The intra-annual SLWB frequency of each Landsat pixel was calculated using the formula (8):

5 $$F(y)=\frac{1}{{N}_{y}}{\sum }_{i=1}^{{N}_{y}}{W}_{y,i}\times 100 \% $$

where *F* is the intra-annual frequency of the water body pixel; *y* is the specified year; *N*_*y*_ is the total number of good Landsat observations of the pixel (no cloud, cloud shadow or snow) in that year; *W*_*y*_,_*i*_ indicates whether the single observation of the pixel is a water body; when *W*_*y*,*i*_ = 1 means water body and W_*y*,*i*_ = 0 means non-water body, and the value range of *F*(*y*) is [0,100%]. Here, five thresholds (0%, 25%, 50%, 75% and 100%) were chosen. To facilitate the analysis of SLWB at different frequencies, we divided SLWB with different frequency thresholds into ephemeral, seasonal and permanent waters^52^. Water pixels with 0% < *F*(*y*) ≤ 25% were classified as ephemeral waters. Water pixels with 25% < *F*(*y*) < 100% were classified as seasonal water bodies and those with *F*(*y*) = 100% were classified as permanent water bodies.

GSW data is frequently used to verify the accuracy of water extraction due to its high precision^28,53^. We utilized the confusion matrix and GSW data to validate the precision of permanent, seasonal and ephemeral water derived from the three algorithms. The results demonstrate that NNE outperforms MNE and IMNE in extracting all three types of water bodies (Tables S2–S4), demonstrating a higher accuracy. These results suggest that NNE can be utilized for further extraction of water body information in time series data.

### Error assessment and temporal trend analysis

Area error, relative area error and area error of the entire study region were calculated with Equations (6–8)^14,54^, respectively.

6 $$Error(1\sigma )=\frac{P}{G}\times \frac{{G}^{2}}{2}\times 0.6872$$

7 $$E=\frac{Error\,(1\sigma )}{A}\times 100 \% $$

where 1*σ* is one standard deviation; *P* is the perimeter of a small lake; *G* is the spatial resolution of the remote sensing image (30 m in this dataset), and 0.6872 is the correction factor at 1*σ*^14,54,55^. *E* is the relative error of the small lake, and *A* is the area of a single small lake.

8 $${E}_{t}=\sqrt{{\sum }_{i=1}^{n}{A}_{i}^{2}}$$

where *E*_*t*_ is the total area error for the entire study area; *i* is the lake number; *n* is the total number of lakes; *A*_*i*_ is the error area for individual lakes^14,54,55^.

We utilized the linear regression method to analyze the interannual variation trends^28^ of permanent and seasonal SLWB areas across different watersheds. Additionally, we conducted a *t*-test to assess the statistical significance^28^ of our findings. The linear regression calculation is shown below:

9 $$Slope=\frac{n\times {\sum }_{i=1}^{n}(i\times {p}_{i})-({\sum }_{i=1}^{n}i)({\sum }_{i=1}^{n}{p}_{i})}{n\times {\sum }_{i=1}^{n}{i}^{2}-{({\sum }_{i=1}^{n}i)}^{2}}$$

where *Slope* is the interannual trend of the permanent or seasonal SLWB area; *n* is the length of time; *i* is the year; and *p*_*i*_ is the permanent or seasonal SLWB area in the year *i*.

## Data Records

The dataset contains 170 vector files stored in ESRI shapefile format for SLWB at different intra-annual water frequency thresholds (0%, 25%, 50%, 75% and 100%) in the QMR from 1987 to 2020. It contains a total of 5 folders, each representing 34 SLWB vector data during 1987‒2020 under a certain threshold. Each vector file contains eight attributes: code, perimeter (km), area (km^2^), latitude and longitude, elevation (m), area error, relative error (%), and subregion. SLWB data^56^ can be downloaded from the data repository Zenodo at 10.5281/zenodo.7392799.

## Technical Validation

### Water extraction algorithm

We improved the MNE equation and proposed NNE. The improvement consisted in changing MNDWI to NDWI in the MNE equation as NDWI outperforms MNDWI in extracting water bodies in the QTP^3,5,57^. In addition, NNE does not include the condition of EVI < 0.1 in the MNE formula, which removes certain water pixels. Additionally, according to our previous study, most vegetation pixels can be effectively removed by (MNDWI > NDVI or MNDWI > EVI)^22^. Our improved NNE offers three advantages. Firstly, common supervised classification algorithms (e.g., neural networks, random forests, support vector machines and object-oriented algorithms) rely on the selection of training samples when extracting water bodies. This study required the extraction of long-term SLWB, and there were challenges such as difficulties in the selection of training samples. NNE can eliminate the need for training sample selection compared to supervised classification, thus avoiding the human subjective error caused by sample selection. Secondly, compared to the common water body index method based on a threshold, NNE does not require a threshold selection, thus avoiding errors in water body extraction due to inaccurate threshold selection. Thirdly, the SLWB extracted using NNE were more extensive compared to MNE and IMNE and avoided incorrect extractions. In summary, our improved NNE algorithm is simple and easy to implement and can be implemented with traditional GIS software (e.g., ENVI, QGIS and ArcGIS) or cloud computing platforms (e.g., GEE and PIE-Engine) for the extraction of water bodies in different regions. Additionally, our method may work better in mountainous areas and needs to be further validated in urban built-up areas. In addition, the data type for our experiments with the SLWB extraction algorithm was Landsat, and the effectiveness of using NNE to extract water bodies based on other types of optical remote sensing data needs to be verified.

### Error assessment for SLWB extraction

**Fig. 6:**
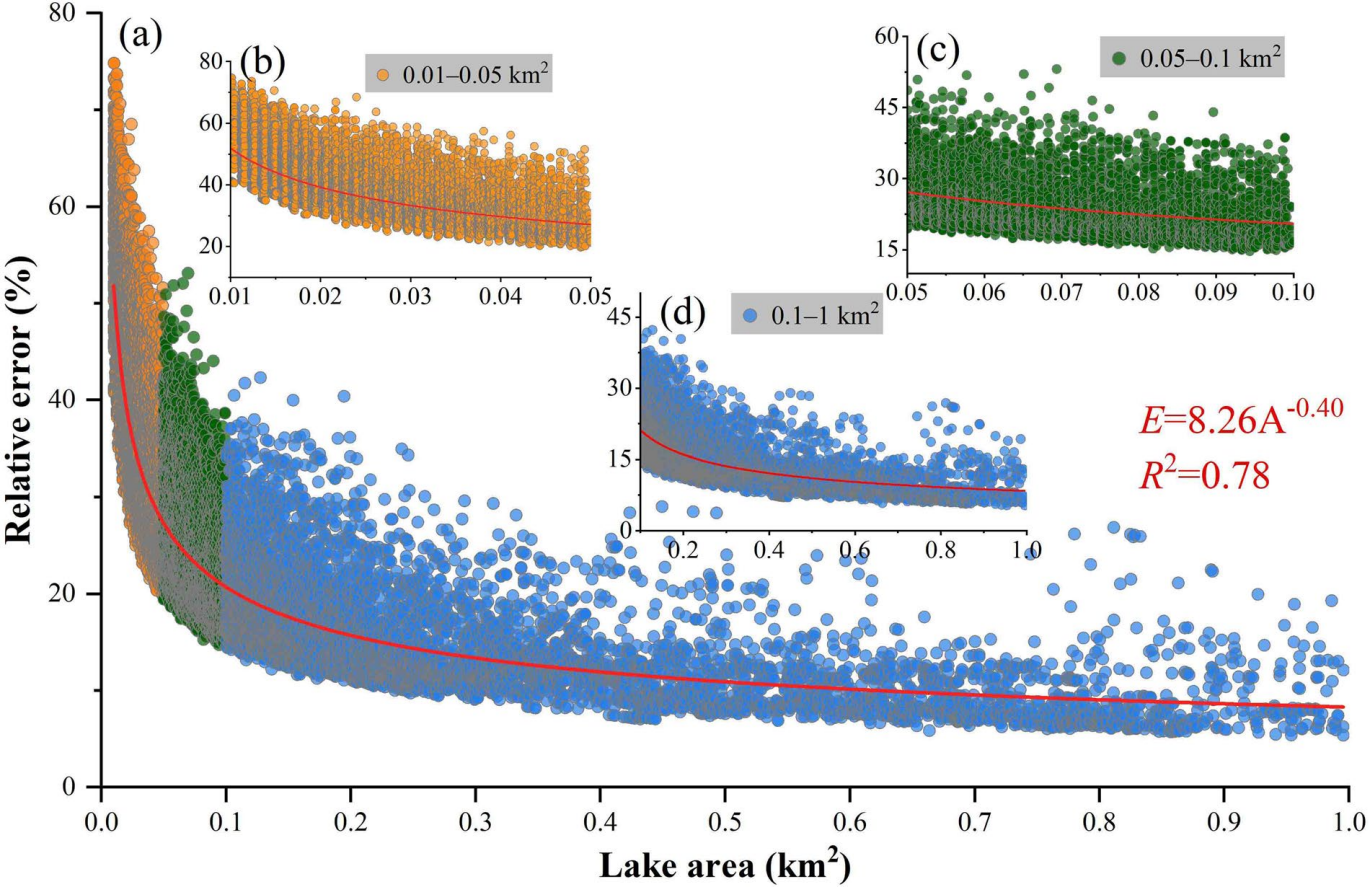
Relationship between size and relative area error of small lakes in the QMR.

The average area of the small lakes during the study period was 0.066 km^2^ and the average perimeter was 1.279 km in the QMR. There was a significant power exponential relationship between the small lake area and relative area error (E = 8.26 A^−0.4^, R^2^ = 0.78, p < 0.001) (Fig. 6). The relative area error of small lakes tended to decrease with increasing size. Similar results were obtained by Wang *et al*.^14^ and Wei *et al*.^54^ in their studies on glacial and thermokarst lakes. The total area of small lakes across the study area for each vector data had an error of ± 0.31 km^2^. We counted the relative area errors for the small lakes and found that the relative errors for all small lakes ranged from 5.39‒74.86%, with an average relative error of 34.49%. The relative area errors for lakes between 0.01‒0.05 km^2^ (accounting for about 24.59% of the total small lake area), 0.05‒0.1 km^2^ (accounting for about 14.59% of the total small lake area) and 0.1‒1 km^2^ (accounting for about 60.82% of the total small lake area) were 40.26%, 23.78% and 15.21%, respectively. Wang *et al*.^14^ drew similar conclusions to ours in their analysis of the glacial lake area errors in high-mountain Asia. Their results suggested that the average relative area errors for small glacial lakes (area ≤ 0.01 km^2^), medium glacial lakes (0.01 km^2^ < area ≤ 0.1 km^2^) and large glacial lakes (area > 0.1 km^2^) were 44.6%, 22.0% and 7.6%, respectively. In addition, Wei *et al*.^54^ also found average relative area errors of 35.1%, 11.4% and 4.4% for the small thermokarst lakes or ponds (area ≤ 0.01 km^2^), medium thermokarst lakes (0.01 km^2^ < area ≤ 0.1 km^2^) and large thermokarst lakes (area > 0.1 km^2^), respectively, when evaluating the accuracy of thermokarst lake extraction in the QTP. This is mainly due to the number of mixed pixels around the edge of an individual lake as a proportion of all the pure lake pixels of the given lake^14,54^. Secondly, the smaller the size of a small lake, the stronger the seasonal variation it tends to exhibit.

### Comparison with other studies

Numerous researchers have studied the large lakes of the QTP^3–5^. Algorithms and theories relating to large lakes have been well established and datasets have been published documenting their area, number, level and volume^58–61^. There have also been some studies on small glacial lakes of the QTP, and some relevant datasets have also been published^14,16^. However, small lakes include not only glacial lakes but also non-glacial lakes, such as artificial lakes and thermokarst lakes formed by the melting of permafrost. Meanwhile, the published datasets on glacial lakes do not take into account seasonal changes in their water bodies over a multi-year period. As a result, the dataset generated in this study could not be directly validated with other existing studies and datasets. However, we indirectly compared it with the datasets published by other researchers^14,16,54,62^.

**Fig. 7:**
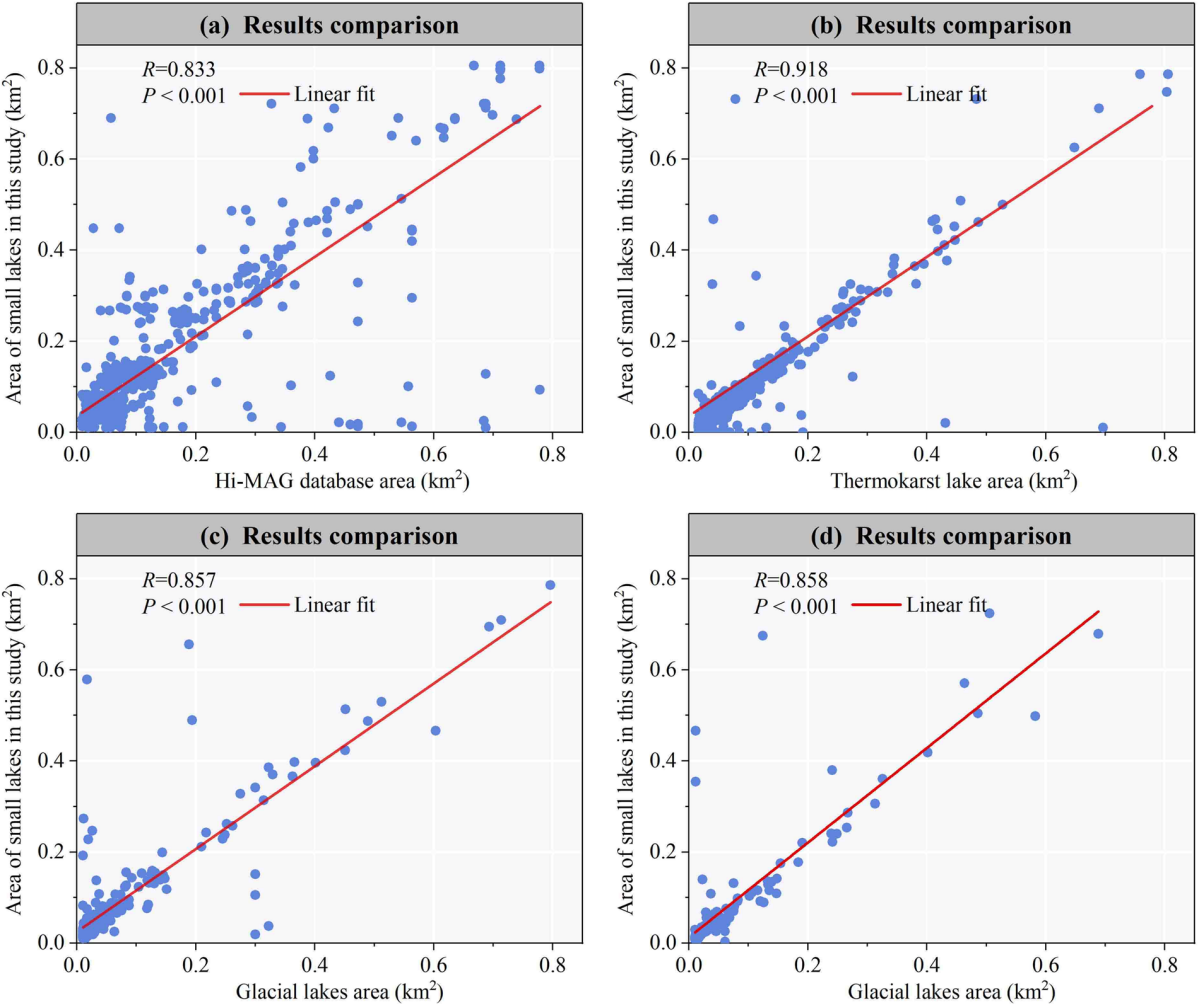
Small lakes area comparisons with Chen *et al*.^16^ (**a**), Wei *et al*.^54^ (**b**), Wang *et al*.^14^ (**c**) and Zheng *et al*.^62^ (**d**).

Chen *et al*.^16^ published an annual 30-m High Mountain Asia glacial-lake inventory (Hi-MAG) dataset^63^ from 2008 to 2017. We selected small lake data from 2008 to 2017 and compared them with the Hi-MAG database. The results show a high agreement between our extracted SLWB and the Hi-MAG dataset, with a correlation coefficient of 0.833 (p < 0.001) (Fig. 7a). However, the small lakes we extracted are larger in area than in the Hi-MAG dataset. This is because of the large differences in the way the two datasets were generated. Our dataset includes small lakes throughout the year, whereas the Hi-MAG dataset shows glacial lakes present at some time between July and November, which would have underestimated some of the small lake areas.

Wei *et al*.^54^ published a dataset of thermokarst lakes and ponds^64^ (500 m^2^‒3 km^2^) in the QTP in 2020. The dataset was generated based on Sentinel-2 data using random forest classification and manual visual interpretation. The accuracy of this dataset has been demonstrated in terms of image spatial resolution and *in situ* measurements. Therefore, it can be used to verify the accuracy of our data. We compared this dataset with the small lakes data we extracted in 2020. The results indicated the highest agreement between small lakes and thermokarst lakes, with a correlation coefficient as high as 0.918 (Fig. 7b).

Wang *et al*.^14^ compiled an Asian alpine glacial lake dataset from 1990 and 2018^65^ that utilized Landsat images. We utilized this dataset to verify the accuracy of the SLWB extracted in this study. The correlation between these two datasets was significant, with a correlation coefficient of 0.857 (p < 0.001) (Fig. 7c). The differences could be attributed to the fact that Wang *et al*.^14^ not only utilized Landsat data from 1990 and 2018 to identify glacial lakes in specific years, but also incorporated data from neighboring periods to improve water body delineation in 1990 and 2018. While the SLWB is subject to change due to various factors, there is a significant margin of error when attempting to extract current-year glacial lake data using Landsat images from neighboring years. Zheng *et al*.^62^ released a glacial lake dataset^66^ covering the Third Pole region from 2014 to 2016, with a spatial resolution of 15 m, including lakes with an area of over 900 m^2^. The correlation analysis demonstrates a high level of agreement between this dataset and the SLWB generated by our study, with a correlation coefficient of 0.858 (p < 0.001) (Fig. 7d).

Zhang *et al*.^5^ found that the large lakes across the QTP experienced a decreasing and then significant increasing trend from the 1990s onwards, which was generally consistent with our findings on small lakes. In addition, recent results from Zhang *et al*.^67^ suggested that all lakes larger than 10 km^2^ in the Qaidanmu Basin of the northeastern QTP experienced first a decreasing and then a significant increasing trend. Moreover, they found that the beginning of the 21st century was an important point in time for changes in the lake area (i.e., there was a significant change since 2000). Our findings also indicated that the area of small lakes across the QMR began to experience a significant change in the year 2000. This indirectly confirms that the dataset we have generated is relatively reliable.

### Limitations and prospects

We have used 13,297 Landsat TM/ETM + /OLI data to extract intra-annual SLWB in the QMR. The published literature and datasets for small lakes (mainly glacial and thermokarst lakes) during the year have been extracted during the summer and autumn (July to November)^14,16^. The relatively low snow cover during this time of year limits its impact on the SLWB extraction. Our study found that both the number and area of intra-annual small lakes in the QMR changed significantly. Therefore, the seasonal effects of small lakes should be considered when studying their spatial and temporal characteristics. Our study provides a reference for the extraction of intra-annual SLWB in other regions.

Small lakes tend to freeze in winter and spring in the QMR when extensive snow cover is present, challenging the extraction of SLWB during this time of year. The NNE algorithm for small lakes in this paper can extract accurate SLWB during non-snowy periods. However, there are still major deficiencies in the boundary extraction of SLWB during the snow accumulation period. How to address the impact of snow on SLWB extraction is a key consideration in our future work.

Extracting SLWB has many challenges and limitations due to the intra-annual water fluctuations in small lakes in the hilly highland regions and the influence of factors such as clouds, cloud shadows and mountain shadows. It is not possible to remove all the poor-quality pixels using Fmask. We used ALOS DSM data to remove mountain shadows. Because of the timing of the acquisition of the ALOS DSM data and the Landsat images used in this study, the calculated topography may not exactly match the actual topography, resulting in minor errors when masking small lakes. Although we corrected these errors in the subsequent manual correction and cross-validation steps, this still introduced some errors in the SLWB extraction.

We have not been able to directly measure the boundaries of typical small lakes in the field using high-precision measuring instruments such as handheld GPS. However, we evaluated the accuracy of three water body extraction algorithms (MNE, IMNE and NNE) using Sentinel-2 data. Sentinel-2 data has also been used to validate the accuracy of the Landsat series data in extracting water bodies^28,53^. This suggests that the Sentinel-2 data can be reliably used for validating the accuracy of Landsat water extractions.

Although this study’s area and number of small lakes were quickly and easily obtainable using satellite remote sensing data, assessing the impacts on water availability requires information on lake basin shapes and shoreline slopes. The magnitude of area variation among different lakes is also not consistent with their water storage variation. In future work, we will select some typical small lakes, conduct field measurements of their basin and establish SLWB and water volume relationships to estimate lake water endowments and improve the storage-area relationships of lakes in different periods; this will allow us to accurately evaluate the relationship between water volume changes and climate change in the lakes of QMR.

### Temporal trends and seasonality of small lakes

**Fig. 8:**
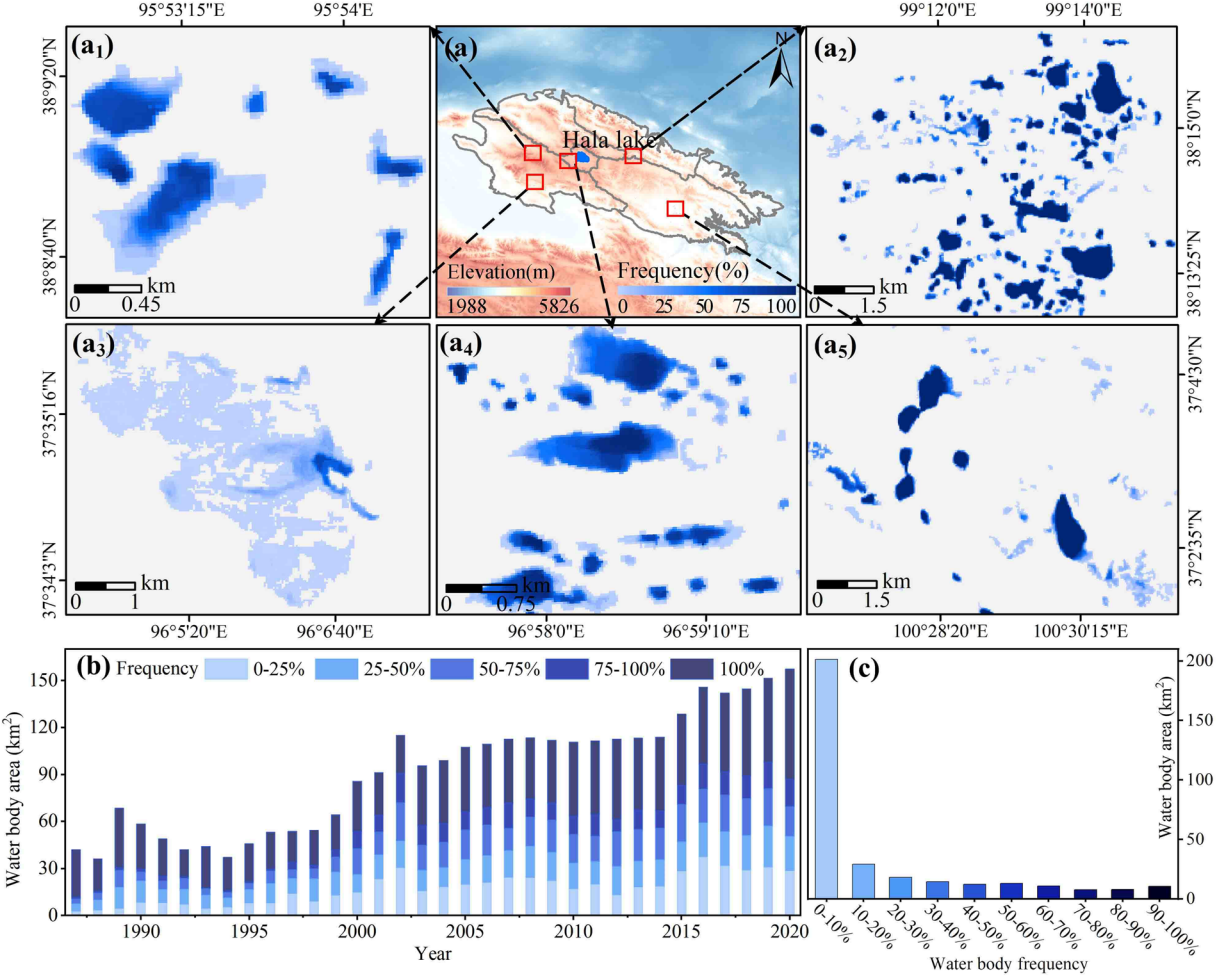
Spatial distribution of SLWB frequency (**a**); Temporal trend of SLWB area (**b**); Frequency distribution of areas of SLWB (**c**).

The spatial distribution of water body frequencies for small lakes in the QMR from 1987 to 2020 was analyzed based on the water body frequency equation. Small lakes were mainly distributed in areas above 4,000 m in elevation (i.e., the western and central parts of the QMR) (Fig. 8a). The SLWB in southern, southwestern and eastern Hala Lake fluctuated largely, mainly due to the higher elevation of these areas, the distribution of glaciers and perennial permafrost, and the relatively high precipitation in these areas. The water bodies with frequencies between 0‒10% (201.49 km^2^) accounted for 61.80% of the SLWB (Fig. 8c). The remaining SLWB frequencies ranged from 7.80‒29.22 km^2^ (2.39‒8.96%) in area. This suggests that the SLWB at the QMR fluctuated relatively heavily from 1987 to 2020 and the proportion of stable water bodies was small. However, there was significant spatial variability in SLWB across basins. The area and proportion of SLWB in each basin in descending order were Qinghai Lake Basin (167.89 km^2^, 67.68%), Hala Lake Basin (29.16 km^2^, 11.76%), Danghe River & Shule River (21.52 km^2^, 8.68%), Beida River & Heihe River Basin (11.66 km^2^, 4.70%), Shiyang River & Datong River Basin (9.17 km^2^, 3.70%), Haleteng River & Bayinguole River Basin (8.63 km^2^, 3.48%).

**Fig. 9:**
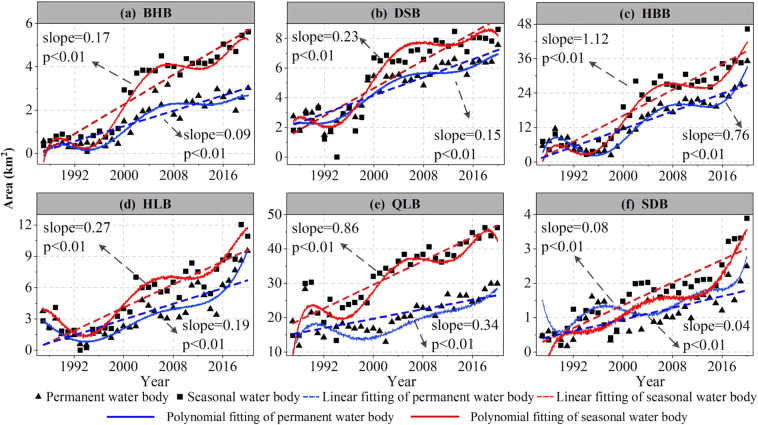
Trends in permanent and seasonal SLWB area from 1987 to 2020 for different basins (QLB: Qinghai Lake Basin; HLB: Hala Lake Basin; SDB: Shiyang River & Datong River Basin; DSB: Danghe River & Shule River; BHB: Beida River & Heihe River Basin; HBB: Haleteng River & Bayinguole River Basin).

The SLWB in the QMR experienced a decreasing and then a significant increasing trend between 1987 and 2020 (Fig. 8b). This is generally consistent with the area trend of large lake water bodies across the QMR, where the area of small lakes has experienced significant changes since 2000. Zhang *et al*.^5^ found that the great lakes (area > 10 km^2^) on the QTP have exhibited a decreasing trend followed by a significant increase over the past 30 years. Furthermore, Wang *et al*.^68^ also indicated that the trends in the lake area (>1 km^2^) in the endorheic basin of the QTP are consistent with the SLWB derived in this study. This further supports the validity of the data and conclusions presented in our study. We further analyzed the temporal trends in permanent and seasonal water of the SLWB for the different catchments of the QMR from 1987 to 2020. The results suggest a significant trend of increasing seasonal and permanent water in six basins (Fig. 9). The Qinghai Lake Basin had the largest seasonal water increase (0.86 km^2^ a^‒1^) and the Shiyang River & Datong River Basin had the smallest (0.08 km^2^.a^‒1^). In terms of trends in permanent water, the largest increase in the Haleteng River & Bayinguole River Basin (0.76 km^2^.a^‒1^) and the smallest increase in the Shiyang River & Datong River Basin (0.04 km^2^.a^‒1^) were observed.

## Supplementary information

Supplementary information

## Data Availability

The lake water extraction for this study was performed on the GEE platform. The GEE JavaScript code can be downloaded at https://github.com/GISLandsat/water-research1.git. GEE should be used to access and edit the code.
