# Supplementary material for: An intra-annual 30-m dataset of small lakes of the Qilian Mountains for the period 1987–2020
Source: Sci Data. 2023 Jun 7;10:365. doi: 10.1038/s41597-023-02285-x (PMC10247696; doi:10.1038/s41597-023-02285-x)
Supplement: Supplementary file 1 — Supplementary information [file 41597_2023_2285_MOESM1_ESM.pdf]

Supporting Information for

An intra-annual 30-m dataset of small lakes of the Qilian Mountains for the period 1987–2020

Chao Li[1,2], Shiqiang Zhang[1,2*], Dahong Zhang[1,2], Gang Zhou[1,2]

[1]College of Urban and Environmental Science, Northwest University, Xi'an 710127, PR China

[2]Shaanxi Key Laboratory of Earth Surface System and Environmental Carrying Capacity, Northwest University, Xi'an 710127, PR China

Corresponding author: Shiqiang Zhang (zhangsq@nwu.edu.cn)

Table of Contents

Table S1. Landsat and Sentinel-2A images for accuracy assessment.

Table S2. Confusion matrix for accuracy assessment of permanent water extraction for three algorithms.

Table S3. Confusion matrix for accuracy assessment of seasonal water extraction for three algorithms.

Table S4. Confusion matrix for accuracy assessment of ephemeral water extraction for three algorithms.

Table S1. Landsat and Sentinel-2A images for accuracy assessment.

**Landsat P&R**<br>**S2 T&N**<br>Date	**131/34**<br>**T48SUG**<br>2020–3–20<br>2020–11–15	**131/35**<br>**T48STF**<br>2020–2–17	**131/35**<br>**T47SQA**<br>2020–2–17	**131/35**<br>**T48SUF**<br>2020–2–17	**132/34**<br>**T47SPA**<br>2020–1–23	**132/35**<br>**T47SPA**<br>2020–1–23<br>2020–12–8
**Landsat P&R**<br>**S2 T&N**<br>Date	**132/33**<br>**T48STH**<br>2020–1–23	**132/33**<br>**T47SQC**<br>2020–1–23	**132/34**<br>**T47SQA**<br>2020–12–8<br>2019–3–9	**132/34**<br>**T48STG**<br>2020–12–8	**132/34**<br>**T48STH**<br>2019–11–4	**132/35**<br>**T47SQA**<br>2019–3–9
**Landsat P&R**<br>**S2 T&N**<br>Date	**132/35**<br>**T48STF**<br>2019–03–9<br>2020–12–8	**132/34**<br>**T48STF**<br>2019–3–9	**132/34**<br>**T47SQB**<br>2020–12–8<br>2019–11–4<br>2020–10–21<br>2018–7–12	**133/33**<br>**T47SPD**<br>2020–10–12<br>2018–10–23	**133/34**<br>**T47SPB**<br>2020–10–12<br>2019–7–22<br>2019–5–3	**133/35**<br>**T47SNV**<br>2020–10–12
**Landsat P&R**<br>**S2 T&N**<br>Date	**133/34**<br>**T47SPA**<br>2020–3–18	**134/33**<br>**T47SMD**<br>2019–4–24	**134/33**<br>**T47SND**<br>2019–4–24	**134/33**<br>**T47SPC**<br>2020–4–10	**134/33**<br>**T47SND**<br>2020–9–17<br>2020–6–29<br>2018–9–28	**135/34**<br>**T47SLB**<br>2018–9–3<br>2017–10–18
**Landsat P&R**<br>**S2 T&N**<br>Date	**135/33**<br>**T47SLD**<br>2018–9–19	**136/33**<br>**T46SGJ**<br>2018–8–25	**136/34**<br>**T46SGG**<br>2018–8–25	**137/33**<br>**T46SFJ**<br>2019–6–16<br>2018–9–17<br>2017–5–25<br>2016–9–27		

Table S2. Confusion matrix for accuracy assessment of permanent water extraction for three algorithms.

Method		Samples	GSW data		Total	User accuracy	Overall accuracy	Kappa coefficient
			Permanent water	Non-permanent water				
MNE	Landsat	Permanent water	4304	13	4317	0.9969	0.9738	0.9470
		Non-permanent water	196	3487	3683	0.9467		
		Total	4500	3500	8000			
	Total producer’s accuracy		0.9564	0.9963				
IMNE	Landsat	Permanent water	4306	13	4319	0.9969	0.9741	0.9476
		Non-permanent water	194	3487	3681	0.9472		
		Total	4500	3500	8000			
	Total producer’s accuracy		0.9568	0.9962				
NNE	Landsat	Permanent water	4309	12	4321	0.9972	0.9746	0.9645
		Non-permanent water	191	3488	3679	0.9480		
		Total	4500	3500	8000			
	Total producer’s accuracy		0.9575	0.9965				

Table S3. Confusion matrix for accuracy assessment of seasonal water extraction for three algorithms.

Method		Samples	GSW data		Total	User accuracy	Overall accuracy	Kappa coefficient
			Seasonal water	Non-seasonal water				
MNE	Landsat	Seasonal water	4152	45	4197	0.9892	0.9508	0.9009
		Non-seasonal water	348	3455	3803	0.9084		
		Total	4500	3500	8000			
	Total producer’s accuracy		0.9226	0.9871				
IMNE	Landsat	Seasonal water	4162	21	4183	0.9949	0.9551	0.9096
		Non-seasonal water	338	3479	3817	0.9114		
		Total	4500	3500	8000			
	Total producer’s accuracy		0.9248	0.9940				
NNE	Landsat	Ephemeral water	4179	20	4199	0.9952	0.9573	0.9140
		Non-ephemeral water	321	3480	3801	0.9155		
		Total	4500	3500	8000			
	Total producer’s accuracy		0.9286	0.9942				

Table S4. Confusion matrix for accuracy assessment of ephemeral water extraction for three algorithms.

Method		Samples	GSW data		Total	User accuracy	Overall accuracy	Kappa coefficient
			Ephemeral water	Non-ephemeral water				
MNE	Landsat	Ephemeral water	4065	89	4154	0.9785	0.9345	0.8683
		Non-ephemeral water	435	3411	3846			
		Total	4500	3500	8000			
	Total producer's accuracy		0.9033	0.9745				
IMNE	Landsat	Ephemeral water	4071	70	4141	0.9830	0.9376	0.8746
		Non-ephemeral water	429	3430	3859			
		Total	4500	3500	8000			
	Total producer's accuracy		0.9046	0.9800				
NNE	Landsat	Ephemeral water	4111	68	4179	0.9837	0.9428	0.8849
		Non-ephemeral water	389	3432	3821			
		Total	4500	3500	8000			
	Total producer's accuracy		0.9135	0.9805				
